# Comparison of Deep Learning Tools for Optic Nerve Axon Quantification Finds Limited Generalizability Upon Independent Validation

**DOI:** 10.3390/bioengineering13060647

**Published:** 2026-05-30

**Authors:** Benton Chuter, Noah Emmert, Min Young Kim, Andrew B. Stiemke, Nikhil Dave, Jay Herrin, Zirui Zhou, Gideon Wall, Lu Lu, Robert W. Williams, Abraham A. Palmer, Hao Chen, T. J. Hollingsworth, Monica M. Jablonski

**Affiliations:** 1 Department of Ophthalmology, Hamilton Eye Institute, University of Tennessee Health Science Center, 930 Madison Avenue, Memphis, TN 38163, USA; mkim80@uthsc.edu (M.Y.K.); ndave1@uthsc.edu (N.D.); jherrin@health.southalabama.edu (J.H.); thollin1@uthsc.edu (T.J.H.); 2 Department of Computer Science and Software Engineering, Miami University, Oxford, OH 45056, USA; nemmert@uthsc.edu; 3 Department of Genetics, Genomics and Informatics, The University of Tennessee Health Science Center, Memphis, TN 38163, USA; drew-s@ameritech.net (A.B.S.); llu@uthsc.edu (L.L.); rwilli10@uthsc.edu (R.W.W.); 4 Frederick P. Whiddon College of Medicine, University of South Alabama, Mobile, AL 36617, USA; 5 College of Medicine, University of Tennessee Health Science Center, Memphis, TN 38163, USA; zzhou24@uthsc.edu (Z.Z.); gwall7@uthsc.edu (G.W.); 6 Department of Psychiatry, University of California San Diego, La Jolla, CA 92093, USA; aapalmer@health.ucsd.edu; 7 Institute for Genomic Medicine, University of California San Diego, La Jolla, CA 92093, USA; 8 Department of Pharmacology, Addiction Science, and Toxicology, The University of Tennessee Health Science Center, Memphis, TN 38163, USA; hchen3@uthsc.edu

**Keywords:** machine learning, deep learning, optic nerve, axon quantification, histology, glaucoma, scoping review, digital pathology, model validation, generalizability

## Abstract

Machine learning approaches for automated quantification of optic nerve histology have emerged as tools for the objective assessment of axonal injury in experimental glaucoma models. However, their generalizability to independent datasets remains unclear. Guided by a scoping review following PRISMA-ScR guidelines, this study evaluated the performance of publicly available models on novel datasets. PubMed, EMBASE, Scopus, and Cochrane CENTRAL were searched (2000–2025). Two reviewers screened studies and extracted model characteristics and performance metrics. Three models (AxoNet, AxonDeepSeg, and AxoNet 2.0) were independently validated on a rat optic nerve dataset (44 images; 6941 axons) and a mouse optic nerve dataset (74 full cross-sections). From 2036 records, four manuscripts describing three deep learning models met the inclusion criteria, with reported correlation coefficients of 0.959–0.99 between model predictions and reference counts. On the rat dataset, the performance correlation declined (r = 0.831–0.907), precision remained high (>0.94), but recall was low (0.18–0.27), with Dice coefficients of 0.29–0.40. On the mouse dataset, correlations decreased further (r = 0.57–0.74) and model rankings differed, reflecting the domain shift and scale-dependent effects. These findings demonstrate strong within-study performance but reduced generalizability to independent datasets, highlighting the need for standardized validation datasets and multi-center testing.

## 1. Introduction

Retinal ganglion cell (RGC) loss represents the defining pathological feature of glaucoma and other optic neuropathies, conditions that together constitute leading causes of irreversible blindness worldwide [1,2,3,4]. The histological quantification of optic nerve axons provides a direct measure of RGC survival and remains essential for evaluating neuroprotective interventions in experimental models. However, manual axon counting is labor-intensive, subject to inter-observer variability, and impractical for the large sample sizes often required in preclinical studies [5,6].

To address these limitations, several approaches have been developed to improve the efficiency and reproducibility of axon quantification. Sampling-based approaches that extrapolate from counted subregions introduce additional variability, while fully manual counts of entire nerve cross-sections require hours per specimen [7,8]. Semi-automated tools such as AxonJ have improved the throughput but still require significant operator input and parameter tuning [9].

Deep learning has transformed digital pathology across medical disciplines, with convolutional neural networks (CNNs) achieving expert-level performance in tumor detection, tissue segmentation, and prognostic classification [10,11,12,13,14,15,16,17]. These methods learn hierarchical feature representations directly from image data, potentially capturing subtle patterns that manual analysis may fail to detect. In ophthalmology, deep learning applications span retinal disease screening, glaucoma detection from fundus photographs, and optical coherence tomography interpretation [18,19].

Despite this progress, machine learning approaches for optic nerve histology have been described in only a limited number of studies, each employing different model architectures, species, staining methods, and outcome measures. This heterogeneity complicates the comparison of model performance and hinders the assessment of which approaches may be most suitable for specific applications. Importantly, the generalizability of these models when applied outside their original training environments remains largely untested. Domain shift, wherein models encounter data distributions differing from their training sets, represents a well-documented challenge in medical imaging that can substantially degrade performance [20,21,22,23,24,25].

This study evaluated the generalizability of published machine learning models for optic nerve axon quantification through independent validation testing guided by a scoping review of the literature. Our objectives were to (1) identify published models and summarize their architectures and training data; (2) synthesize reported performance metrics across studies; (3) identify gaps in the current evidence base; and (4) evaluate model performance on independent datasets from BXD mice and outbred rat optic nerve images not used in prior training or validation.

## 2. Materials and Methods

### 2.1. Scoping Review Design

The scoping review was conducted in accordance with the Preferred Reporting Items for Systematic Reviews and Meta-Analyses extension for Scoping Reviews (PRISMA-ScR) guidelines [26]. A scoping review methodology was selected given the anticipated heterogeneity across studies and our objective of mapping the breadth of available evidence rather than synthesizing pooled effect estimates. The protocol was registered with the International Prospective Register of Systemic Reviews (PROSPERO) prior to screening (registration: CRD420251250152).

#### 2.1.1. Eligibility Criteria

Studies were eligible for inclusion if they met the following criteria: (1) involved human or animal optic nerve tissue, retinal ganglion cells, axons, myelin, or associated glial cells; (2) applied machine learning methods for quantification, segmentation, or morphometric analysis of histological images; (3) reported quantitative performance metrics comparing automated outputs to reference standards; and (4) were published as peer-reviewed original research articles written in English between 2000 and 2025 (Supplementary Table S1).

Studies were excluded if they did not involve optic nerve or related structures; used only traditional histologic grading without machine learning components; lacked quantitative outcomes; or were reviews, editorials, conference abstracts without full data, or non-peer-reviewed publications.

#### 2.1.2. Information Sources and Search Strategy

Four electronic databases were searched: PubMed; EMBASE; Scopus; and Cochrane CENTRAL. The search strategy combined three concept blocks using Boolean operators. The first block captured anatomical terms (optic nerve, retinal ganglion cell, axon, myelin, astrocyte, microglia, oligodendrocyte, glia, and fibroblast). The second block captured computational methods (deep learning, machine learning, convolutional neural network, CNN, segmentation, and automated). The third block captured histological analysis (histology, histopathology, histomorphometry, morphometry, quantification, and grading). The complete search strategies for each database are provided in Supplementary Table S2.

#### 2.1.3. Selection Process

**Figure 1:**
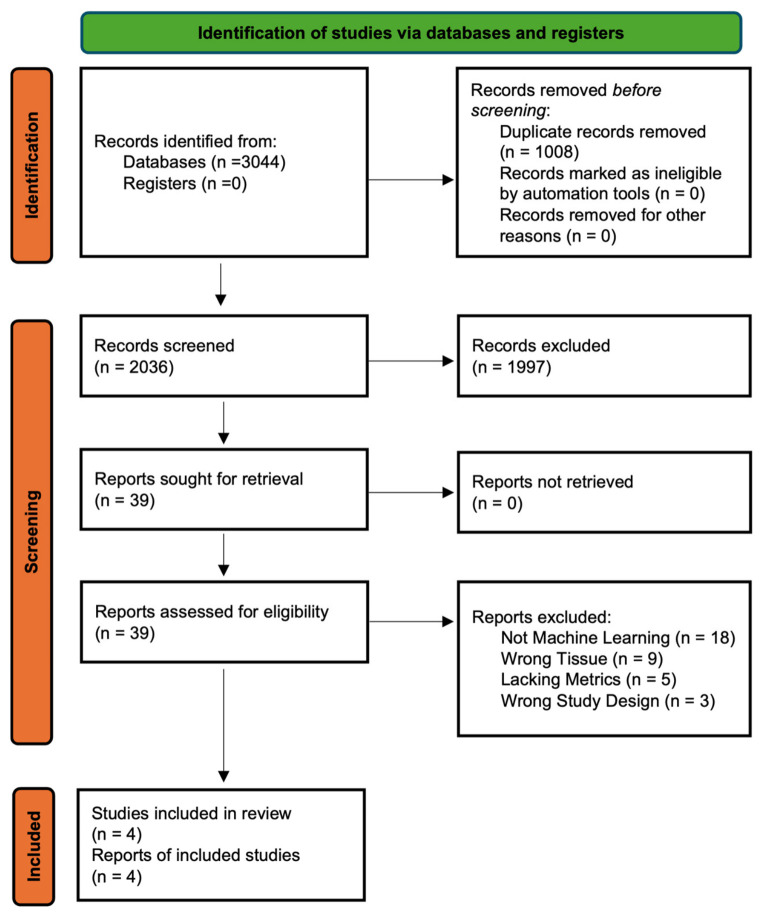
PRISMA 2020 flow diagram for the scoping review [27]. Of 2036 unique records screened across PubMed, EMBASE, Scopus, and Cochrane CENTRAL, 39 underwent full-text review, and 4 manuscripts describing 3 distinct deep learning models (AxoNet, AxonDeep, and AxoNet 2.0) met inclusion criteria. Per-stage exclusion reasons are shown in the diagram.

Two reviewers independently screened titles and abstracts against predefined eligibility criteria. Records deemed potentially relevant by either reviewer advanced to full-text review. Full-text articles were independently assessed by both reviewers. Disagreements were resolved through structured discussion based on the eligibility criteria and rationale for inclusion or exclusion. If consensus could not be reached, a third reviewer adjudicated the decision. The selection process was documented using the PRISMA flow diagram (Figure 1).

#### 2.1.4. Data Extraction

A standardized data extraction form was developed and piloted on two studies prior to full extraction. Two reviewers independently extracted data from each included study, with discrepancies resolved by consensus. Complete extracted data are provided in Supplementary Table S3. Extracted variables included: study characteristics (authors, year, and institution); model characteristics (name, architecture type, and training approach); dataset characteristics (species, strain, disease model, staining method, and sample sizes); and performance metrics (correlation coefficients, Dice coefficients, mean absolute error, root mean squared error, and Bland–Altman limits of agreement).

### 2.2. Independent Validation Study

To assess model generalizability beyond published performance metrics, we conducted independent validation testing of available models on novel datasets from rat and mouse samples. The validation dataset from heterogenous stock rats comprised 44 paraphenylenediamine (PPD)-stained optic nerve cross-section sub-images (256 × 256 pixel tiles, 0.055 micrometers per pixel) containing 6941 manually annotated axons that were prepared using our previously described methods [28]. Axon annotations were performed using COCO Annotator v0.11.1 software by annotators SG, KF, SP, and WE. All annotations were reviewed and adjudicated by ABS to ensure consistency across the dataset.

Additional validation was conducted using the mouse dataset comprising 74 PPD-stained full mouse optic nerve cross-sections from archival tissue collected across multiple experimental conditions. Each mouse cross-section contained 10,000 to 45,000 manually annotated axons with a mean of 21,443 axons per nerve. Manual axon annotations were performed by annotator ABS. The archival nature of this tissue, with older imaging protocols and more variable tissue quality, represents a more challenging test condition than standardized prospective imaging. Axon counts from multiple graders were reported as mean ± SEM, which were used as our ground truth dataset for both rat and mouse models.

Three models were evaluated: AxoNet (using the final_resampled_3-22-2020.hdf5 checkpoint), AxonDeepSeg (using the model_seg_generalist_BF_light configuration) [29], and AxoNet 2.0 (using the standard U-Net architecture). Because AxonDeep [30] is not publicly available, direct independent validation of AxonDeep could not be performed. Therefore, we evaluated AxonDeepSeg as a publicly available alternative for neural network-based axon segmentation. AxonDeepSeg was developed for general axon and myelin segmentation from microscopy data and represents a distinct implementation from AxonDeep, though both employ deep learning for axon identification. Accordingly, comparisons between AxonDeep metrics should be interpreted cautiously.

All models were applied using their published implementations and default parameters without any fine-tuning or adaptation to the validation dataset. The intrinsic preprocessing pipelines of each model were left intact: AxoNet and AxoNet 2.0 perform per-image rescaling of grayscale inputs to the unit interval without stain or histogram normalization, while AxonDeepSeg (model_seg_generalist_BF_light) applies per-image z-score normalization through the underlying nnU-Net framework. As a sensitivity analysis to assess whether the observed performance differences reflect intrinsic model limitations or remediable distributional mismatch, we additionally evaluated each model on validation images that had been histogram-matched to a reference image drawn from the corresponding training distribution. Out-of-the-box and histogram-matched results are compared in Supplementary Figures S1 and S2. The same three models were applied to the mouse dataset using identical implementations and default parameters, adjusted only to reflect pixel-scale differences between the datasets.

Performance was assessed at two levels. For axon count prediction accuracy, we computed Pearson correlation coefficients (r), Spearman’s rank correlation coefficients (ρ), Lin’s concordance correlation coefficient (CCC), mean absolute error (MAE), root mean squared error (RMSE), and systematic bias between model-predicted and ground truth axon counts for each image. Pearson correlation quantifies linear association, whereas Lin’s CCC evaluates both precision and accuracy into a single agreement index [31,32]. MAE and RMSE quantify the magnitude of error irrespective of direction, whereas systematic bias was calculated as the mean signed difference between predicted and reference counts, reflecting the direction of error (over- or undercounting) on average.

Agreement bias and dispersion were further evaluated using Bland–Altman 95% limits of agreement (LoA) calculated from paired count differences [33], with strength-of-agreement interpretation. The Shapiro–Wilk test and Q-Q plot inspection were used to assess normality of paired differences and determine whether parametric LoA (mean bias ± 1.96 SD) or non-parametric LoA (2.5th and 97.5th percentiles of differences) should be reported. Ninety-five percent confidence intervals for all performance metrics, including Pearson r, Lin’s CCC, Spearman ρ, MAE, RMSE, and Bland–Altman bias, were computed using the bias-corrected and accelerated (BCa) bootstrap with 10,000 resamples [34]. Per-image paired predictions, additional agreement metrics with BCa 95% CIs, and the normality assessment are reported in Supplementary Tables S5 and S6 and Figure S3.

For segmentation quality, we computed pixel-level metrics by comparing predicted segmentation masks to ground truth annotations. The Dice coefficient and intersection over union (IoU) quantify spatial overlap between predicted and reference segmentations (range: 0–1), with Dice placing greater emphasis on shared regions and IoU comparing overlap to the total combined area. Precision represents the proportion of predicted axon pixels that are correct, while recall represents the proportion of true axon pixels correctly identified. Because the mouse dataset included only total axon counts without individual axon segmentation masks, evaluation was limited to count-based metrics.

#### Performance Metrics

An important consideration for comparing performance across studies is the dependence of certain metrics on evaluation tile size of sub-image patches used for analysis. Correlation coefficients, MAE, RMSE, and systematic bias all vary with tile dimensions because smaller tiles restrict the range of axon counts per tile, which can inflate correlation values. Metrics that are normalized to count magnitude, such as mean absolute percentage error (MAPE) and relative bias, are not affected by tile size and allow for comparison across different evaluation scales. Similarly, pixel-level segmentation metrics (Dice coefficient, IoU, precision, and recall) are computed as ratios and are not dependent on tile dimensions. Among the studies identified in this review, none reported MAPE, relative bias, or any other count-normalized metric for axon quantification (Supplementary Table S4). Ninety-five percent confidence intervals for all performance metrics were computed using non-parametric bootstrap resampling with 10,000 replicates and the bias-corrected and accelerated (BCa) method [34,35].

### 2.3. Data Synthesis

Given the scoping nature of the review and the heterogeneity across studies, a narrative synthesis approach was employed for published results. Study and model characteristics were summarized descriptively in tables, and performance metrics were presented graphically. For metrics reported by multiple studies, weighted means were calculated using sample size weights. Independent validation results were analyzed separately and compared to published benchmarks to quantify generalizability gaps.

## 3. Results

### 3.1. Scoping Review

#### 3.1.1. Study Selection

The initial database search identified 3044 records across PubMed, EMBASE, Scopus, and Cochrane CENTRAL. After the removal of duplicates, 2036 unique records underwent title and abstract screening. Of these, 1997 were excluded for not meeting predefined eligibility criteria, including studies not clearly specific to optic nerve tissue and those using non-fully automated or semi-automated quantification approaches. The remaining 39 articles underwent full-text review, of which 35 were excluded: 18 did not apply machine learning methods; nine did not involve optic nerve tissue; five lacked quantitative performance metrics; and three were review articles. Four manuscripts describing three distinct deep learning models met all inclusion criteria and were included in the final synthesis (Figure 1).

#### 3.1.2. Characteristics of Included Studies

The included studies were published between 2020 and 2023, originating from research groups in the United States. All studies focused on the automated quantification of retinal ganglion cell axons in optic nerve cross-sections from experimental glaucoma models. Table 1 summarizes model characteristics and presents published performance metrics.

AxoNet was described by Ritch et al. (2020) and evaluated on two datasets: a rat dataset comprising 27 optic nerves from 14 Brown Norway rats with unilateral experimental glaucoma (1% toluidine blue staining; 1514 annotated sub-images) [36], and a non-human primate (NHP) dataset from Reynaud et al. (2012) consisting of optic nerves from rhesus and cynomolgus macaques (PPD staining; 494 annotated sub-images) [8].

AxonDeep was described by Deng et al. (2021) [30] and applied to optic nerves from 56 mice across three strains: DBA/2J, D2.Lyst, C57BL with blast-induced traumatic brain injury (C57BL/6J), and Diversity Outbred mice (C57BL/J:DO). The dataset included 78 sub-images with PPD staining [30].

AxoNet 2.0 was described in two publications by Goyal et al. [31,37], evaluating the model on Brown Norway rats with unilateral ocular hypertension. The primary dataset included 46 eyes from 23 rats (1% toluidine blue staining; 1421 annotated sub-images). The model was also validated on DBA/2J mice with blast-induced traumatic brain injury and on rhesus and cynomolgus macaque optic nerves, demonstrating cross-species applicability.

#### 3.1.3. Model Architectures

All three models employed convolutional neural network architectures based on encoder–decoder designs (Table 1). AxoNet uses a U-Net style architecture that outputs pixelwise axon count density estimates rather than binary segmentation masks. AxonDeep employs a semi-supervised learning framework that integrates a fully convolutional segmentation network with a discriminator network in a generative adversarial network (GAN) architecture. In this approach, the discriminator learns to distinguish model-generated segmentations from expert annotations, providing an additional training signal that reduces the need for fully labeled data. AxoNet 2.0 refined the original AxoNet architecture with improved training procedures and data augmentation strategies. Because the original AxonDeep model was not publicly available, independent validation was performed using AxonDeepSeg [29], a related U-Net-based architecture for axon and myelin segmentation.

#### 3.1.4. Published Performance Metrics

**Figure 2:**
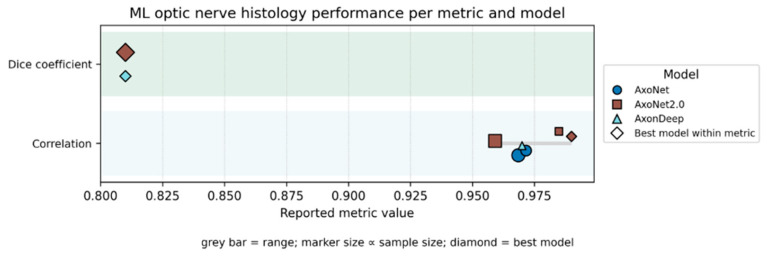
Forest plot of published Pearson r across included models. Marker size scales with sample size; gray bars span the range across model–dataset combinations (absent when only one value was reported); and diamonds mark the best performer per metric.

Reported performance metrics varied across studies, with correlation coefficients being the most consistently reported measure (Table 1, Figure 2). All three models demonstrated strong agreement with manual reference counts in their original publications. For our study, published correlation coefficients (r or r-squared) across six models–datasets were converted to r for comparison, which ranged from 0.959 to 0.99.

AxoNet achieved r = 0.97 on the rat testing subset and r = 0.97 on the NHP testing subset. AxonDeep reported r = 0.97 between semi-supervised predictions and reference counts. AxoNet 2.0 achieved the highest published correlation with r = 0.99 on mouse optic nerves.

Segmentation accuracy was reported as the Dice coefficient in two models. Both AxonDeep and AxoNet 2.0 reported Dice coefficients of 0.81, indicating substantial overlap between the predicted and reference segmentation masks.

Mean absolute error was only reported for AxoNet: 4.4 axons per sub-image on the rat testing subset, and 17.7 axons per sub-image on the NHP testing subset. AxonDeep reported a mean absolute percentage error of 4.4% for the mouse model. The mean squared error was reported for AxoNet 2.0 with 6.18, 63.16, and 74.71 axons for the rat, mouse, and NHP models, respectively.

### 3.2. Independent Validation Results on Outbred Rat Optic Nerve

**Figure 3:**
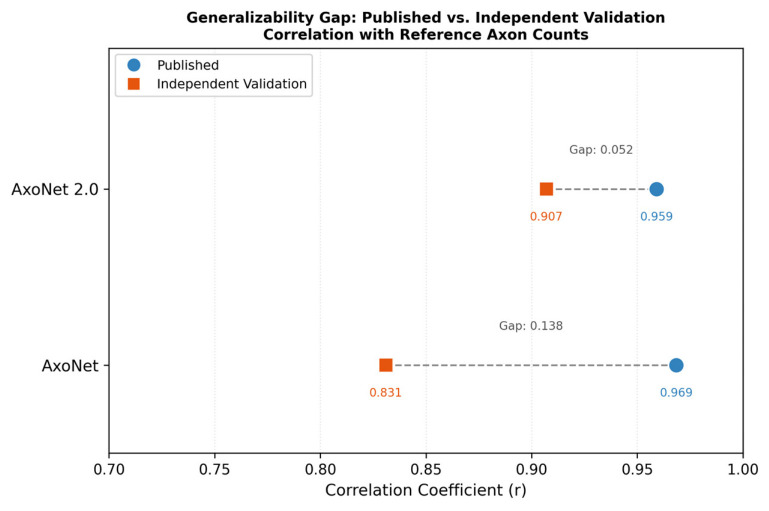
Pearson r for predicted vs. manual axon counts: published values (blue circles) vs. independent rat validation (red squares; n = 44 images not used in any model’s training). Dashed lines connect paired values; gap magnitude is in parentheses. AxonDeepSeg is omitted (no published correlation benchmark).

Independent validation across deep learning models on the novel rat optic nerve dataset generated in our laboratory, with known manual axon counts (n = 44 images, 6941 axons), revealed performance differences from published benchmarks (Table 2, Figure 3). As described in the Methods section, AxonDeepSeg was evaluated in place of the publicly unavailable AxonDeep implementation.

#### 3.2.1. Axon Count Agreement

All models maintained positive correlations with ground truth axon counts, though at reduced levels compared to published values. AxoNet 2.0 achieved the highest correlation (r = 0.907, MAE = 63.2 axons, and RMSE = 74.9 axons), followed by AxonDeepSeg (r = 0.899, MAE = 75.0 axons, and RMSE = 90.3 axons) and AxoNet (r = 0.831, MAE = 113.7 axons, and RMSE = 133.2 axons). The correlation decrements from published values ranged from 0.052 points (AxoNet 2.0) to 0.138 points (AxoNet).

#### 3.2.2. Segmentation Quality

Pixel-level segmentation metrics revealed a consistent pattern across models: high precision but low recall (Table 2). AxoNet 2.0 achieved Dice = 0.40, IoU = 0.26, precision = 0.94, and recall = 0.27. AxonDeepSeg achieved Dice = 0.29, IoU = 0.18, precision = 0.95, and recall = 0.18. These Dice coefficients were substantially lower than the published benchmark of 0.81, representing decrements of 0.41 to 0.52 points.

**Figure 4:**
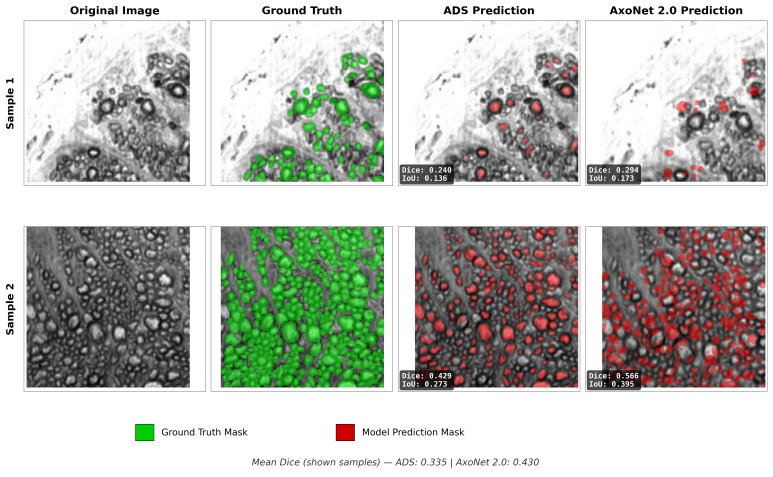
Representative examples of axon segmentation. Representative examples of AxonDeepSeg (ADS) and AxoNet 2.0 axon segmentation on independent rat optic nerve tests are shown.

The high precision values (>0.94) indicate that when models identified pixels as belonging to axons, they were usually correct. However, the low recall values (0.18 to 0.27) indicate that models detected only a fraction of the total axon area present in ground truth annotations (Figure 4).

#### 3.2.3. Findings

**Figure 5:**
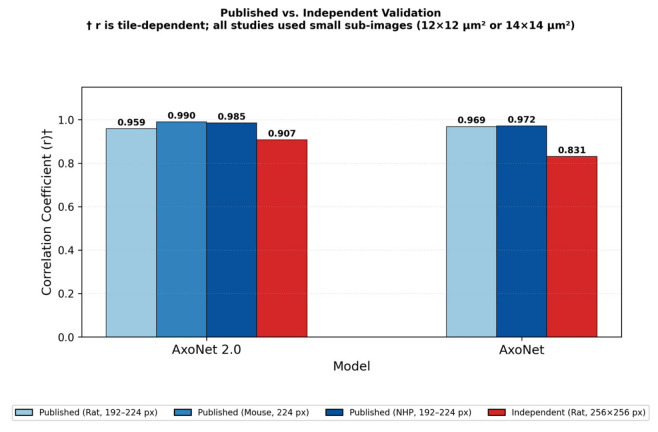
Axon count agreement (Pearson r) between model predictions and manual reference counts. Blue indicates published benchmark values from original model publications, and red indicates independent validation performance (rat, n = 44 images).

Comparison of published and independent validation results reveals a consistent generalizability gap (Figure 3). Published correlation coefficients clustered between 0.96 and 0.97, while independent validation correlations ranged from 0.831 to 0.907. The magnitude of this gap varied by model, with AxoNet 2.0 showing the smallest performance decrement and AxoNet the largest. A detailed comparison of published versus independent validation metrics is provided in Figure 5. Although Pearson r remained high on the rat dataset (0.831 to 0.907; BCa 95% CI 0.699 to 0.949), Lin’s concordance correlation coefficients were substantially lower (CCC 0.169 to 0.649; Supplementary Table S5), reflecting systematic undercounting (Bland–Altman bias of −63.2 to −113.7 axons per 256 by 256 px patch on a mean ground truth of 158 axons) that the linear correlation does not penalize but agreement metrics, segmentation recall (0.18 to 0.27), and Dice (0.29 to 0.40) all do.

Notably, model rankings differed between published results and independent validation. AxoNet achieved the highest published correlation for rat models (r = 0.97) but had the lowest correlation in independent validation (r = 0.831) among the three models. Although AxonDeep reported a similar published correlation (r = 0.97), it could not be directly evaluated, because its implementation is not publicly available; instead, the alternative model AxonDeepSeg achieved r = 0.899 upon independent validation. In contrast, AxoNet 2.0 reported a lower published correlation for rat models (r = 0.96) but achieved the highest correlation in independent validation performance (r = 0.907).

### 3.3. Independent Validation Results on BXD Mouse Optic Nerve

Additional validation was performed on 74 full mouse optic nerve cross-sections from archival tissue from our laboratory, representing a qualitatively different evaluation condition from both published benchmarks and the primary rat optic nerve dataset. Unlike the tile-level evaluation used in published studies and the rat dataset (~12 to 14 um^2^ tiles containing 5 to 50 axons), each mouse image comprised a complete optic nerve cross-section containing approximately 10,000 to 45,000 axons.

**Figure 6:**
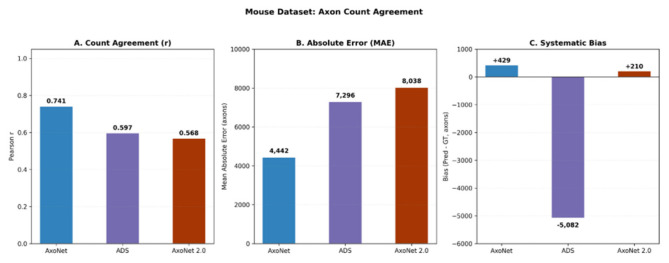
Performance of three models on 74 full BXD mouse optic nerve cross-sections (PPD-stained archival tissue; mean ground truth = 21,443 axons, range ~10,000–45,000). (**A**) Pearson r [BCa 95% CI]: AxoNet 0.741 [0.598–0.861], ADS 0.597 [0.361–0.774], and AxoNet 2.0 0.568 [0.391–0.692]. (**B**) Mean absolute error. (**C**) Systematic bias (mean signed error). All correlations fell below published benchmarks and the rat dataset, consistent with a greater domain shift.

**Figure 7:**
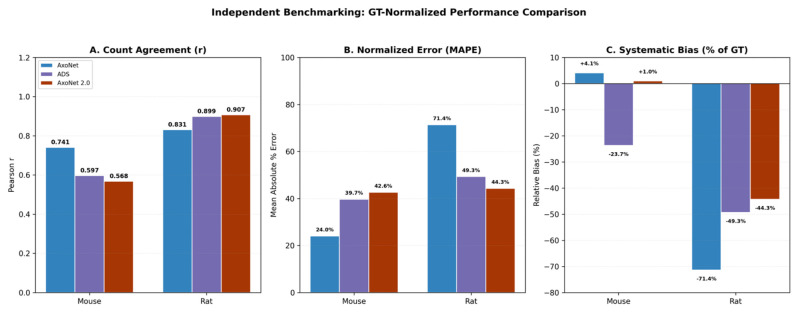
Ground truth-normalized performance on the BXD mouse (n = 74 full cross-sections, mean GT = 21,443 axons) and outbred rat (n = 44 patches, 256 × 256 px, and mean GT = 158 axons) datasets, enabling comparison across count magnitudes. (**A**) Pearson r. (**B**) Mean absolute percentage error (MAPE). (**C**) Mean relative bias (% of GT).

AxoNet achieved the highest correlation (r = 0.741, MAE = 4443 axons), followed by AxonDeepSeg (r = 0.597, MAE = 7296 axons) and AxoNet 2.0 (r = 0.568, MAE = 8038 axons; Figure 6). Systematic bias analysis revealed that AxonDeepSeg substantially undercounted (bias = −5082 axons), while AxoNet and AxoNet 2.0 demonstrated modest overcounting (bias = +429 and +210 axons, respectively). Notably, model rankings differed from both the rat dataset and published benchmarks, with AxoNet performing best despite having the lowest correlation on rat tiles (Figure 7).

Lower correlations on mouse tissue likely reflect both differences in the data characteristics and evaluation scale. The archival nature of the mouse tissue, including variation in imaging protocols and species differences, introduces a domain shift relative to training conditions. In addition, because ground truth counts for the mouse dataset are whole-nerve stereological estimates without per-tile annotations, the mouse data cannot be retrospectively tiled to match the published evaluation scale. These results therefore represent what happens when models trained and evaluated on small tiles are applied to entire optic nerve cross-sections: there is a domain shift in both tissue characteristics and evaluation scale.

### 3.4. Tile-Size Dependency of Reported Metrics

Examination of evaluation methodology across included studies revealed that all published counting performance metrics are dependent on the dimensions of the image tiles used for evaluation. All included published studies evaluated models on small sub-image tiles: AxoNet used 192 × 192 pixel tiles (~12 × 12 μm^2^), AxoNet 2.0 used 224 × 224 pixel tiles (~12 × 12 μm^2^), and AxonDeep used 192 × 192 pixel tiles of comparable physical scale. Our primary independent validation on optic nerve images from rats used 256 × 256 pixel tiles (~14 × 14 μm^2^), a similar physical scale that permits a relatively fair comparison of correlation coefficients with published values.

In contrast, evaluation on the mouse dataset was performed on full optic nerve cross-sections containing approximately 10,000 to 45,000 axons per image, representing evaluation units that are orders of magnitude larger than the published tiles containing approximately 5 to 50 axons each. Because correlation is sensitive to the range of the underlying variable, the lower mouse r values (0.57 to 0.74) reflect both the true performance variation and a systematic deflation from the wider count range, making direct comparison with published tile-level correlations misleading.

No published study reported tile-independent counting metrics (MAPE or relative bias), precluding any tile-size-invariant comparison between published and independently validated performances (Supplementary Table S4).

## 4. Discussion

In this study, we identified three previously published deep learning models for optic nerve axon histology quantification through a scoping review and conducted the first independent validation to compare their performance on a novel dataset. Although all models demonstrated a strong agreement with reference standards in their original publications, with correlation coefficients exceeding 0.96, independent validation revealed meaningful performance decrements ranging from 0.052 to 0.138 correlation points. These findings have important implications for the adoption and further development of automated optic nerve histology tools.

### 4.1. The Generalizability Gap

The observed performance decrements on independent validation align with broader patterns in medical imaging, where deep learning models frequently show a degraded performance when applied to data from different sources than their training sets [11,12]. In optic nerve histology, potential sources of domain shift include differences in tissue preparation protocols, staining intensity and consistency, image acquisition parameters, and anatomical variation across species and strains [32,33,38,39].

To distinguish intrinsic model limitations from remediable intensity–distribution mismatch, we conducted a sensitivity analysis applying histogram matching to align validation images to a representative training distribution image prior to inference (Supplementary Figures S1 and S2). Performance under histogram matching for rat data improved by Δr = 0.08 for AxoNet, decreased by Δr = 0.03 for AxonDeepSeg, and showed minimal change (0.01) for AxoNet 2.0. The mouse dataset showed the opposite pattern: AxoNet declined (Δr = −0.06), AxonDeepSeg improved (Δr = +0.04), and AxoNet 2.0 declined substantially (Δr = −0.08). The inconsistency of direction and magnitude across datasets indicates that the generalizability gap is driven by multiple factors beyond intensity–distribution mismatch and is not reliably remediable by simple histogram alignment. The asymmetric impact across models is consistent with their differing intrinsic preprocessing: AxonDeepSeg, which performs per-image z-score normalization through nnU-Net, demonstrated less sensitivity to histogram matching than AxoNet and AxoNet 2.0, which do not perform comparable normalization. These results reinforce the conclusion that out-of-the-box deployment yields meaningful performance decrements that simple preprocessing cannot fully mitigate.

Additional validation on a second independent dataset (mouse, n = 74) showed correlations of r = 0.57 to 0.74, lower than on the rat dataset (r = 0.831 to 0.907). Two factors contribute to this difference. First, the archival mouse tissue, with older imaging protocols and more variable quality, introduces a greater degree of domain shift from training conditions. Second, the mouse evaluation was performed at the whole-nerve level (10,000 to 45,000 axons per image) rather than the tile level (~5 to 50 axons), and correlation is systematically deflated when computed over a wider count range. These two effects are confounded and cannot be separated without a per-tile ground truth for the mouse dataset, which does not exist. The reversal in model rankings between datasets demonstrates that performance depends on the relationship between training data and the target domain, including the evaluation scale, and that published benchmarks should not be assumed to transfer across tissue sources or evaluation conditions.

The magnitude of the generalizability gap varied by model, suggesting that certain architectural or training choices may confer greater robustness [40]. On the rat dataset, AxoNet 2.0 exhibited the smallest performance decrement (0.052 correlation points), possibly reflecting its more extensive training data or refined augmentation strategies. AxoNet showed the largest decrement (0.138 points) despite being trained on rat tissue, similar to our validation dataset. Such a difference may be attributed to AxoNet’s density estimation model, which may systematically fail to recognize small axons even with high correlation. While correlation values from the mouse dataset were analyzed (Supplementary Figure S2), with AxoNet achieving the highest correlation, these values are not directly comparable to tile-based evaluations due to differences in the evaluation scale. Together, these findings demonstrate that even same-species validation cannot guarantee generalizability across laboratories.

### 4.2. Segmentation Quality Versus Count Agreement

The apparent contradiction between a high Pearson correlation (r = 0.83 to 0.91 on rat tiles) and low pixel-level segmentation quality (Dice 0.29 to 0.40; recall 0.18 to 0.27) reflects the different properties measured by these metrics. The Pearson correlation rewards a linear relationship between predicted and reference counts regardless of whether predictions cluster around the identity line. In contrast, Dice and recall evaluate pixel-level segmentation accuracy. A model that systematically under-segments axons can therefore still achieve a high Pearson correlation if the undercounting remains approximately proportional across images. Lin’s CCC penalizes departures from the identity line and therefore captures both correlation and bias. On the rat dataset, CCC values dropped sharply for two of the three models. AxoNet demonstrated the greatest discrepancy (Pearson r = 0.831 [BCa 95% CI 0.699 to 0.903]; CCC = 0.169 [0.121 to 0.227]), quantitatively confirming that the strong rank-order agreement masks systematic undercounting of approximately 70% of the per-tile axon count (mean signed bias of −113.7 axons against a ground truth mean of 158 axons; Supplementary Table S5). The same systematic undercounting is reflected in the low pixel-level recall and Dice scores, since axons absent from the predicted mask are simultaneously absent from the count. Together, these findings demonstrate that the Pearson correlation alone can overestimate the agreement with reference counts in the regime where systematic bias is large, whereas CCC, Bland–Altman bias, and pixel-level metrics provide a more complete assessment of model performance.

Segmentation masks were not available for the mouse dataset. A notable finding from independent validation on the rat dataset was the dissociation between segmentation quality and count agreement. Despite achieving reasonably strong correlations for axon counts (r = 0.79 to 0.89), segmentation metrics revealed substantial limitations. Dice coefficients of 0.29 to 0.40 fell well below the published benchmark of 0.81, driven primarily by low recall values (0.18 to 0.27). This pattern, characterized by high precision (>0.94) and low recall, indicates conservative segmentation: models correctly identified axon pixels when they did so but missed substantial portions of the true axon area. This suggests that models may be under-detecting axon boundaries or missing smaller axon profiles. Consistent with this interpretation, the rat dataset demonstrated substantial negative bias across models, indicating systematic undercounting. For applications focused solely on axon counting, this may be acceptable if under-segmentation is consistent across images. However, for morphometric analyses requiring accurate axon size measurements, this conservative bias could systematically underestimate axon diameters and areas.

In contrast, bias in the mouse dataset was more variable, with some models showing modest overcounting. One possible explanation is the presence of degenerating axons in archival tissue, which may retain abnormal or partially preserved structural features despite the loss of normal axonal integrity, as described in studies of optic nerve degeneration [41,42]. Such structurally ambiguous features may be classified as intact axons by automated methods, contributing to overestimation. This highlights that both the direction and magnitude of bias are influenced by underlying tissue characteristics, not solely the model architecture.

### 4.3. Evaluation Scale and Metric Selection

An underappreciated factor in the interpretation of model performance is the dependence of counting metrics on evaluation tile size. Therefore, performance comparisons across studies using different evaluation units, such as tiles versus whole cross-sections, are not directly comparable and lead to misinterpretation of the model performance.

In our study, lower correlations observed in the mouse dataset (r = 0.57 to 0.74) cannot be interpreted as a proportionally worse performance, as the evaluation was performed on full optic nerve cross-sections. The lower mouse correlations reflect a combination of genuine performance variation due to the domain shift and a measurement artifact from the scale mismatch.

The absence of tile-independent counting metrics in the published literature limits our ability to make fully scale-invariant comparisons. None of the three included studies reported the MAPE or relative bias for axon counting (Supplementary Table S4), metrics that would allow for fair comparison regardless of tile size. Although we computed these metrics for our independent validation datasets (Table 2), the lack of published baselines precludes direct comparison. This represents a reporting limitation in the current literature. Future studies should report both tile-dependent (r, MAE) and tile-independent (MAPE, relative bias) metrics to enable valid cross-study and cross-scale comparisons.

### 4.4. Performance in Context

Despite the observed generalizability gap, the independent validation correlations of 0.831 to 0.907 remain potentially useful for many research applications. These values compare favorably to the inter-observer variability reported for manual counting methods, which can exceed 10 to 15 percent of the coefficient of variation [7,43]. The key consideration is whether the reduced accuracy is acceptable for specific experimental questions and whether systematic biases might confound treatment group comparisons.

The published Dice coefficients of 0.81 reported by both AxonDeep and AxoNet 2.0 warrant reconsideration in light of our independent validation findings. These values were obtained on held-out test sets drawn from the same data sources as the training sets, representing within-distribution testing. Our observed Dice coefficients of 0.29 to 0.40 are likely to better reflect the expected performance in typical research settings where models encounter novel tissue preparations.

### 4.5. Limitations

Several limitations should be considered when interpreting these findings. First, our primary independent validation used a single dataset from one laboratory (rat, n = 44), though additional validation on a second dataset (mouse, n = 74) showed consistent patterns. Although bootstrap confidence intervals were reported for all performance metrics to quantify uncertainty at the image level, external multi-center validation remains necessary to establish broader reproducibility. Generalizability of the validation results to other tissue types, species, or imaging protocols remains to be established. Second, the validation dataset for both axon count and segmentation metrics was limited to rat optic nerve tissue with PPD staining; performance on other species or staining methods was not assessed. Third, we evaluated models using their published implementations without fine-tuning, representing an out-of-the-box performance that could potentially be improved with dataset-specific adaptation. Fourth, because AxonDeep is not publicly available, we could not directly validate this model and instead tested AxonDeepSeg as a publicly available alternative, which is an independently developed tool from a different research group with a different architecture and training paradigm. AxonDeepSeg results should therefore be interpreted as an evaluation of that tool’s applicability to optic nerve histology, not as a proxy for AxonDeep performance.

The scoping review itself has limitations. All included studies originated from a small number of research groups, potentially limiting the diversity of approaches. The absence of studies applying machine learning to human optic nerve histology limits clinical translation. Heterogeneity in outcome reporting prevented direct meta-analytic synthesis.

Finally, comparability of performance across evaluation scales is limited. Confounding of the domain shift and evaluation scale in the mouse dataset cannot be disentangled without per-tile ground truth annotations, which were not available.

### 4.6. Future Directions

Several developments would strengthen the evidence base for machine learning in optic nerve histology. Multi-center validation studies using standardized datasets are needed to assess generalizability across laboratories and establish realistic performance expectations. Shared benchmark datasets with expert-consensus annotations would enable the direct comparison of models and support the development of domain adaptation techniques.

Standardization of reporting practices would improve comparison across studies. We recommend that future studies report correlation coefficients, mean absolute error, and Dice coefficients using consistent definitions, along with sample sizes and confidence intervals. Because correlation and absolute error metrics are dependent on the evaluation tile size, studies should also report tile-independent counting metrics, particularly MAPE and relative bias, to enable valid comparison across different evaluation scales and between laboratories using different imaging and tiling parameters. Validation on held-out data from external sources should become standard practice before model publication.

Investigation of domain adaptation and transfer learning approaches may help bridge the generalizability gap. Techniques such as few-shot learning, unsupervised domain adaptation, and self-supervised pretraining have shown promise in other medical imaging domains and warrant exploration for optic nerve histology [44]. Public release of model implementations would also facilitate independent validation and broader adoption.

## 5. Conclusions

Current deep learning models for optic nerve axon histology achieve a strong agreement with expert reference counts in within-study evaluations, with published correlation coefficients exceeding 0.96. However, independent validation reveals meaningful performance decrements when models are applied to novel datasets, with correlations ranging from 0.831 to 0.907 and segmentation Dice coefficients from 0.29 to 0.40. This generalizability gap demonstrates the importance of external validation before widespread adoption. Among tested models, AxoNet 2.0 demonstrated the most consistent performance on independent validation. Evaluation on a second independent dataset further confirmed that these performance decrements are not dataset-specific, with correlations of 0.57 to 0.74 on archival mouse optic nerve tissue. Future work should prioritize multi-center validation studies, standardized benchmark datasets, public release of model implementations, and the development of domain adaptation techniques to improve model generalizability across laboratories and tissue preparations.

## Figures and Tables

**Table 1:** Characteristics and reported performance metrics of deep learning models for optic nerve axon quantification.

Model	Author/Year	Architecture	Training	Staining	Species	Sample Size	Reported Correlation	Dice	MAE	RMSE
AxoNet	Ritch et al., 2020 [36]	U-Net (density)	Supervised	Toluidine blue	Brown Norway rat	1514 sub-images	R^2^ = 0.938	-	4.4	-
				PPD	NHP *	494 sub-images	R^2^ = 0.944	-	17.7	-
AxonDeep	Deng et al., 2021 [30]	GAN-based FCN	Semi-supervised	PPD	Mouse (DBA/2J, D2.Lyst, C57BL/6J and J:DO)	78 sub-images (56 optic nerves)	R = 0.97	0.81	4.4% **	-
AxoNet 2.0	Goyal et al., 2023a, 2023b [31,37]	U-Net (refined)	Supervised	Toluidine blue	Brown Norway rat	1421 sub-images	R^2^ = 0.92	0.81	-	6.18
				PPD	DBA/2J Mouse ***	22 sub-images	R^2^ = 0.98	-	-	63.16
					NHP *	50 sub-images	R^2^ = 0.97	-	-	74.71

Abbreviations: GAN = generative adversarial network framework; FCN = fully convolutional network; PPD = p-paraphenylenediamine; NHP = non-human primate; DO = Diversity Outbred; MAE = mean absolute error (axons per sub-image); and RMSE = root mean squared error. Correlation values represent R^2^ for AxoNet models and Pearson R for AxonDeep. * Species not explicitly named in Ritch et al. (2020) [36] or Goyal et al. (2023b) [37]; NHP datasets were annotated from Reynaud et al. (2012) [8] for rhesus and cynomolgus macaques from the Burgoyne Lab (Devers Eye Institute). ** Mean absolute percentage error was reported in this study. *** Mouse dataset was annotated from Deng et al. (2021) [30].

**Table 2:** Independent validation results on the outbred rat and BXD mouse datasets for three deep learning axon counting models.

Dataset	Model	Pearson r [95% CI]	Lin’s CCC [95% CI]	Spearman Rho [95% CI]	MAE [95% CI]	RMSE [95% CI]	Bias [95% CI]	Bland–Altman LoA	Tile Area (µm^2^)	px/µm	Approx Axons/ Image	MAPE (%)	Rel. Bias (%)	RMSPE (%)	Dice	IoU	Precision	Recall
Outbred Rat (n = 44)	AxoNet	0.831 [0.699, 0.903]	0.169 [0.121, 0.227]	0.854 [0.727, 0.927]	113.7 [94.2, 135.3]	133.2 [111.8, 156.5]	−113.7 [−135.3, −94.2]	[−259.8, −23.8]	196	18.28	~160	71.36	−71.36	83.25	-	-	-	-
	AxonDeepSeg	0.899 [0.837, 0.939]	0.478 [0.398, 0.559]	0.922 [0.843, 0.965]	75 [61.0, 90.8]	90.3 [74.8, 107.0]	−75.0 [−90.8, −61.0]	[−171.4, −9.2]	196	18.28	~160	49.32	−49.32	56.44	0.29	0.18	0.95	0.18
	AxoNet 2.0	0.907 [0.828, 0.949]	0.649 [0.528, 0.751]	0.909 [0.832, 0.955]	63.2 [52.5, 76.6]	74.9 [62.8, 92.9]	−63.2 [−76.6, −52.5]	[−139.5, −13.5]	196	18.28	~160	44.29	−44.29	46.81	0.4	0.26	0.94	0.27
BXD Mouse (n = 74)	AxoNet	0.741 [0.598, 0.861]	0.724 [0.591, 0.836]	0.66 [0.488, 0.783]	4442.5 [3685.5, 5315.4]	5666.3 [4811.5, 6680.1]	428.6 [−858.2, 1744.0]	[−10,721.1, 11,578.4]	varies	2.7	~18,000	24.02	4.07	30.63	N/A			
	AxonDeepSeg	0.597 [0.361, 0.774]	0.478 [0.311, 0.659]	0.506 [0.266, 0.682]	7296.2 [6203.2, 8504.4]	8902.8 [7763,10,249]	−5081.6 [−6696.1, −3385.0]	[−19,507.1, 9343.8]	varies	2.7	~18,000	39.69	−23.69	48.12				
	AxoNet 2.0	0.568 [0.391, 0.692]	0.496 [0.348, 0.626]	0.525 [0.326, 0.680]	8038.1 [6903.6, 9236.9]	9521.3 [8412,10,772]	209.9 [−1918.9, 2400.8]	[−18,574.8, 18,994.5]	varies	2.7	~18,000	42.62	1	51.47				

Abbreviations: Pearson r = correlation between predicted and ground truth counts; CCC = concordance correlation coefficient; CI = confidence interval; LoA = limits of agreement; MAE = mean absolute error; RMSE = root mean squared error; bias = mean signed difference (positive = overcounting, negative = undercounting); MAPE = mean absolute percentage error (per-image error normalized by ground truth count); Rel. bias = mean relative bias as percentage of ground truth; and IoU = intersection over union. The mouse dataset evaluation tile comprises full optic nerve cross-sections (mean ground truth = 21,443 axons), while the rat dataset comprises 256 × 256 px patches (mean ground truth = 158 axons). GT-normalized metrics (MAPE, Rel. bias) allow for direct comparison across datasets with different count magnitudes. Lin’s concordance correlation coefficient (CCC), Spearman’s rho, Bland–Altman limits of agreement, and bias-corrected and accelerated (BCa) bootstrap 95% confidence intervals (10,000 resamples) for all metrics in this table are provided in Supplementary Table S5; the accompanying Shapiro–Wilk normality assessment of paired differences is in Supplementary Table S6 and Supplementary Figure S3.

## Data Availability

A detailed summary of study characteristics and reported performance metrics is available in Table S3. Analysis code and independent validation dataset supporting this review are available from the corresponding author upon reasonable request. Model implementations used for independent validation are publicly available from their original repositories: AxoNet and AxoNet 2.0 (https://github.com/VidishaGoyal/AxoNet-2.0, Accessed 1 January 2026) and AxonDeepSeg (https://github.com/axondeepseg, Accessed 1 January 2026).

## Supplementary Materials

The following supporting information can be downloaded at: https://www.mdpi.com/article/10.3390/bioengineering13060647/s1. Figure S1: Predicted versus ground truth axon counts on the outbred rat optic nerve tiles for AxoNet, AxonDeepSeg, and AxoNet 2.0 across three preprocessing conditions; Figure S2: Ground truth normalized performance metrics for the three models on the outbred rat and BXD mouse independent validation datasets under each preprocessing condition; and Figure S3: Q-Q plots of paired count differences for the three models on the outbred rat and BXD mouse independent validation datasets. Table S1: Scoping review inclusion and exclusion criteria; Table S2: Database search strategies; Table S3: Complete data extraction for included studies; Table S4: Tile-size dependency of performance metrics; Table S5: Mean of complete analysis metrics for both outbred rat and BXD mouse datasets; Table S6: Shapiro–Wilk normality assessment of paired count differences supporting the choice of parametric versus non-parametric Bland–Altman limits; Table S7: Sensitivity analysis of preprocessing alignment strategies on independent validation performance on outbred rat and BXD mouse datasets.

## Abbreviations

The following abbreviations are used in this manuscript:

RGC	Retinal ganglion cell
CNN	Convolutional neural networks
PRISMA-ScR	Preferred Reporting Items for Systematic Reviews and Meta-Analyses extension for Scoping Reviews
PROSPERO	International Prospective Register of Systemic Reviews
PPD	Paraphenylenediamine
MAE	Mean absolute error
RMSE	Root mean squared error
IoU	Intersection over union
MAPE	Mean absolute percentage error
NHP	Non-human primate
GAN	Generative adversarial network
