# Supplementary material for: Comparison of Deep Learning Tools for Optic Nerve Axon Quantification Finds Limited Generalizability Upon Independent Validation
Source: Bioengineering (Basel). 2026 May 30;13(6):647. doi: 10.3390/bioengineering13060647 (PMC13295766; doi:10.3390/bioengineering13060647)
Supplement: Supplementary file 1 [file bioengineering-13-00647-s001.zip › bioengineering-4315628-supplementary.pdf]

SUPPLEMENTARY MATERIALS

Supplementary Figure S1. Scatter plots of predicted versus ground truth axon counts on rat optic nerve tiles for AxoNet, AxonDeepSeg, and AxoNet 2.0 across three preprocessing conditions (unmatched, intensity matched, and Reinhard color normalized).

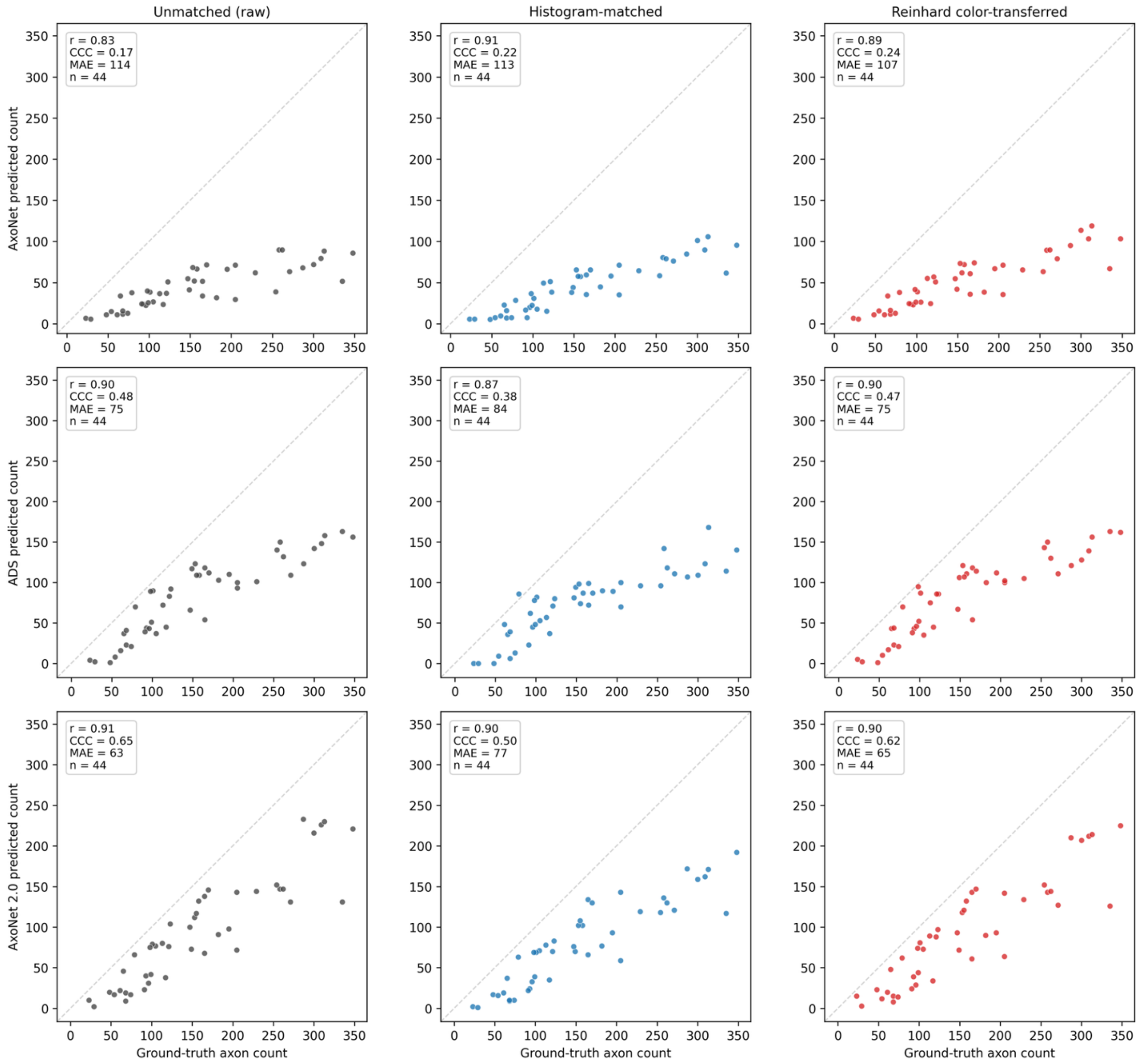

Supplementary Figure S2. Performance of AxoNet, AxonDeepSeg, and AxoNet 2.0 expressed as ground truth normalized metrics on the rat and mouse independent validation datasets.

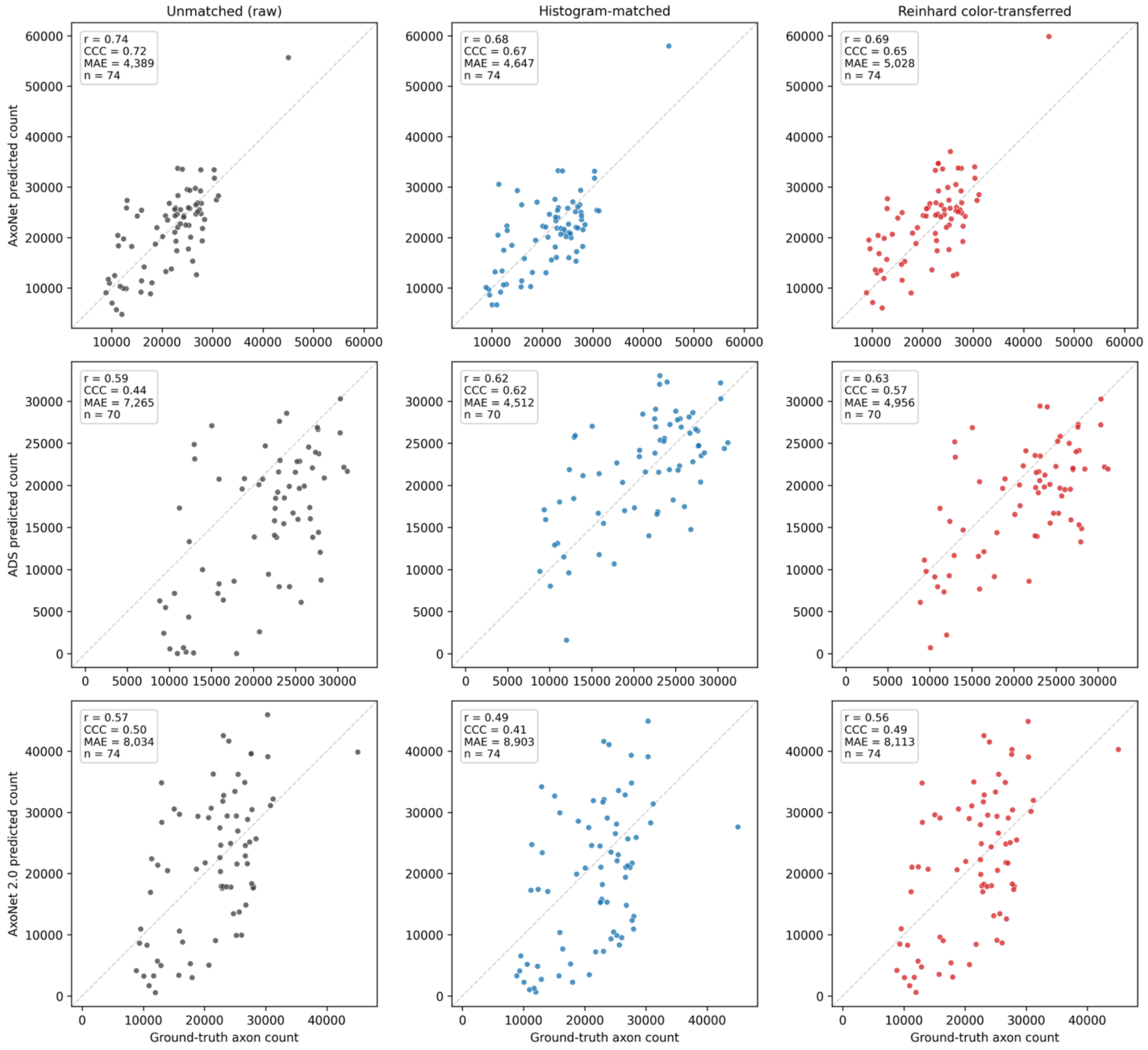

Supplementary Figure S3. Q-Q plots of paired count differences (prediction minus ground truth) on the rat and mouse independent validation datasets, with the diagonal indicating the normal reference.

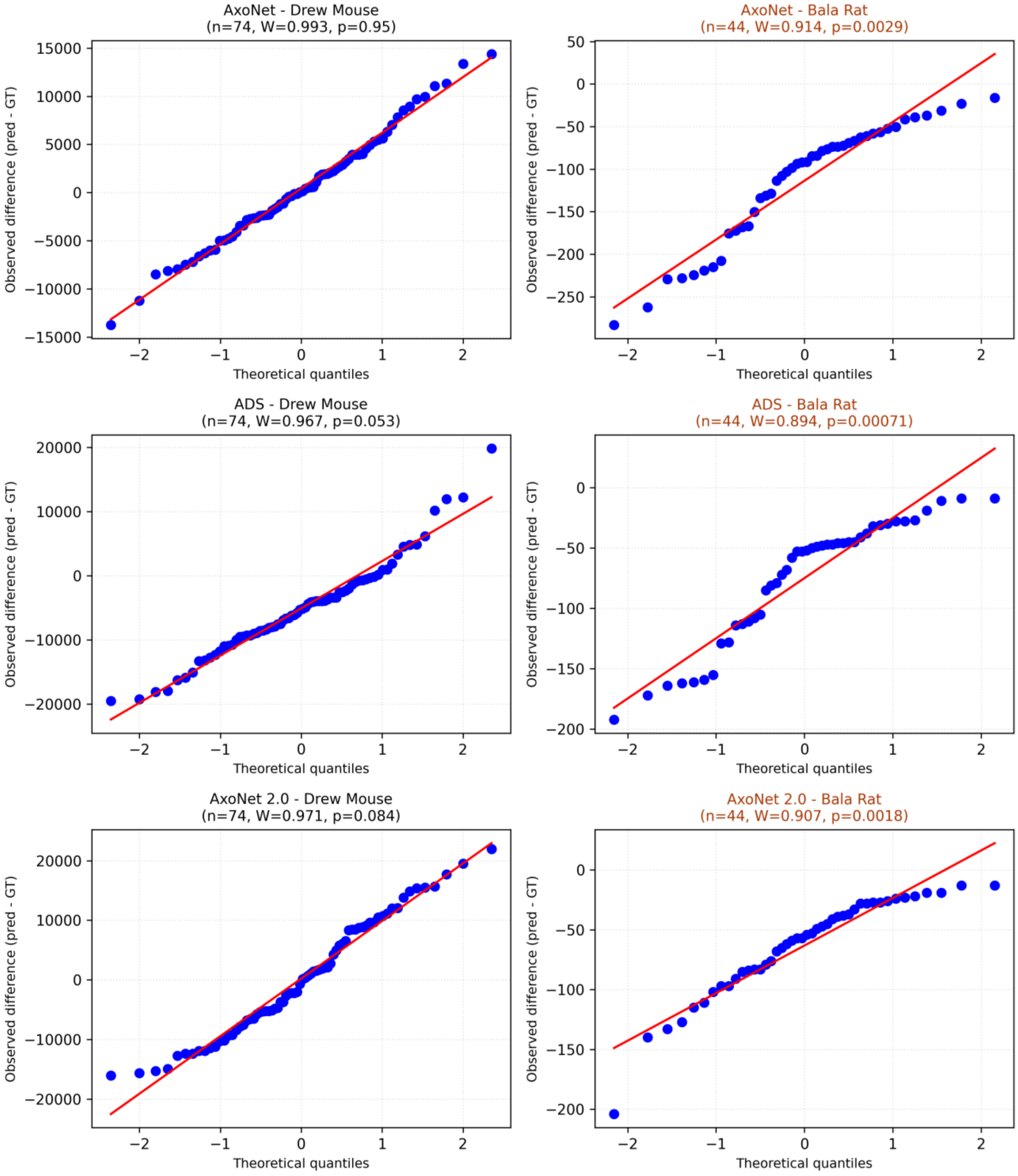

Supplementary Table S1: Scoping Review Inclusion and Exclusion Criteria

Category	Inclusion Criteria	Exclusion Criteria
Population / Sample	Studies involving human or animal optic nerve tissue, retinal ganglion cells, axons, myelin, astrocytes, microglia, oligodendrocytes, or fibroblasts	Studies excluding the optic nerve or related glial/axonal structures (e.g., purely cortical, spinal cord, or non-ocular tissues)
Intervention / Method	Use of machine learning (ML) methods for quantification, segmentation, morphometry, or histopathology analysis of optic nerve/glial tissue, where ML is understood to be any computational method that learns a mapping from input data to output predictions through optimization of model parameters	Studies limited to traditional histologic grading/manual counting without ML/DL components.
Outcomes	Reporting quantitative outcomes such as axon/myelin counts, morphometry, segmentation accuracy, detection performance, or comparative metrics (e.g., Dice score, sensitivity/specificity)	Studies without quantitative outcomes, or descriptive-only pathology papers without algorithmic analysis.
Study Type	Original research articles (basic science, translational, preclinical, or clinical)	Reviews, editorials, commentaries, conference abstracts without full data, non-peer-reviewed gray literature.
Language	English	Non-English
Publication Date	2000-2025 (to capture modern ML/DL applications)	Studies published prior to 2000 (pre-modern ML)

Abbreviations: DL = deep learning; ML = machine learning.

Supplementary Table S2. Database Search Strategies

Database	Search Strategy
PubMed	("optic nerve"[Title/Abstract] OR "retinal ganglion cell*"[Title/Abstract] OR axon*[Title/Abstract] OR myelin[Title/Abstract] OR astrocyte*[Title/Abstract] OR microglia*[Title/Abstract] OR oligodendrocyte*[Title/Abstract] OR glia[Title/Abstract] OR fibroblast*[Title/Abstract]) AND ("deep learning"[Title/Abstract] OR "machine learning"[Title/Abstract] OR "convolutional neural network"[Title/Abstract] OR CNN[Title/Abstract] OR segmentation[Title/Abstract] OR automated[Title/Abstract]) AND (histology[Title/Abstract] OR histopathology[Title/Abstract] OR histomorphometry[Title/Abstract] OR morphometry[Title/Abstract] OR quantification[Title/Abstract] OR grading[Title/Abstract])
EMBASE	('optic nerve':ti,ab OR 'retinal ganglion cell*':ti,ab OR axon*:ti,ab OR myelin:ti,ab OR astrocyte*:ti,ab OR microglia*:ti,ab OR oligodendrocyte*:ti,ab OR glia:ti,ab OR fibroblast*:ti,ab) AND ('deep learning':ti,ab OR 'machine learning':ti,ab OR 'convolutional neural network':ti,ab OR CNN:ti,ab OR segmentation:ti,ab OR automated:ti,ab) AND (histology:ti,ab OR histopathology:ti,ab OR histomorphometry:ti,ab OR morphometry:ti,ab OR quantification:ti,ab OR grading:ti,ab)
Scopus	(TITLE-ABS-KEY("optic nerve") OR TITLE-ABS-KEY(axon*) OR TITLE-ABS-KEY(myelin) OR TITLE-ABS-KEY(astrocyte*) OR TITLE-ABS-KEY(microglia*) OR TITLE-ABS-KEY(oligodendrocyte*) OR TITLE-ABS-KEY(glia) OR TITLE-ABS-KEY(fibroblast*) OR TITLE-ABS-KEY(retinal AND ganglion AND cell*)) AND (TITLE-ABS-KEY("deep learning") OR TITLE-ABS-KEY("machine learning") OR TITLE-ABS-KEY("convolutional neural network") OR TITLE-ABS-KEY(CNN) OR TITLE-ABS-KEY(segmentation) OR TITLE-ABS-KEY(automated)) AND (TITLE-ABS-KEY(histology) OR TITLE-ABS-KEY(histopathology) OR TITLE-ABS-KEY(histomorphometry) OR TITLE-ABS-KEY(morphometry) OR TITLE-ABS-KEY(quantification) OR TITLE-ABS-KEY(grading))
Cochrane CENTRAL	("optic nerve" OR "retinal ganglion cell*" OR axon* OR myelin OR astrocyte* OR microglia* OR oligodendrocyte* OR glia OR fibroblast*) AND ("deep learning" OR "machine learning" OR "convolutional neural network" OR CNN OR segmentation OR automated) AND (histology OR histopathology OR histomorphometry OR morphometry OR quantification OR grading)

Searches conducted in January 2025. Date limits: 2000-2025. Language: English.

Supplementary Table S3. Complete Data Extraction for Included Studies

Summary of study characteristics, model specifications, and reported performance metrics extracted from the four manuscripts meeting inclusion criteria. All studies focused on deep learning approaches for automated quantification of retinal ganglion cell axons in optic nerve cross-sections from experimental models.

**Study Characteristics**	**Author/ Year**	Ritch et al., 2020 [17]		Deng et al., 2021 [16]	Goyal et al., 2023a [18]			Goyal et al., 2023b [19]
	**Institution**	Georgia Institute of Technology; Emory University; Atlanta VA Healthcare System		University of Iowa	Georgia Institute of Technology; Emory University; Atlanta VA; University of Iowa; Legacy Research Institute			Georgia Institute of Technology; Emory University; Atlanta VA Healthcare System
**Model Characteristics**	**Model**	AxoNet		AxonDeep	AxoNet 2.0			
	**Architecture/ Training**	U-Net encoder-decoder; outputs pixelwise axon count density estimates; Supervised		ResNeXt-50 encoder with feature pyramid decoder; semi-supervised GAN framework	VGG16-based U-Net-style encoder-decoder CNN; soft Dice-loss optimization; Supervised			VGG16-based U-Net-style encoder-decoder CNN; soft Dice-loss optimization; Supervised; soft Dice loss optimization
**Dataset Characteristics**	**Optic Nerve Staining**	Toluidine blue	PPD	PPD	Toluidine blue	PPD		Toluidine blue
	**Species**	Rat	NHP* (rhesus and cynomolgus macaque)	Mouse	Rat	Mouse**	NHPs* (rhesus and cynomolgus macaque)	Rat
	**Strain/ Disease Model**	Brown Norway; Unilateral glaucoma induced by anterior-chamber injections	Control and glaucoma eyes	DBA/2J (various degrees of an inherited age-related form of glaucoma), D2.B6-*Lyst*^{bgJ}/Andm (D2.*Lyst*; healthy), C57BL/6J (subjected to blast-induced TBI) and J:DO (Diversity Outbred with healthy but natural variability from genetic background)	Brown Norway; unilateral ocular hypertension induced by anterior chamber injection	DBA/2J; inherited glaucoma and blast wave-induced traumatic optic neuropathy	Glaucoma induced unilaterally by trabecular network laser treatment	Brown Norway; Unilateral ocular hypertension induced by anterior chamber injection
	**Sample Size**	27 ONs from 14 rats; 1,514 sub-images	ONs from 30 eyes; 494 sub-images	56 ONs from 56 mice; 78 sub-images	46 eyes from 23 rats; 1,421 sub-images	ONs from 22 mice; 22 sub-images	ONs from 25 NHPs; 50 sub-images	Cohort IV: 9 ONs from 9 normotensive eyes; Cohort V: 68 eyes from 34 rats with unilateral

**Performance Metrics and Limitations**								ocular hypertension; Cohort VI: 33 eyes from 23 rats with unilateral ocular hypertension
	**Reference Standard**	Manual axon annotations by trained observers		Manual segmentations and center markings by expert annotators	Pixelwise annotations by two masked human annotators combined into probabilistic maps			Morrison grading scale (MGS) for semi-quantitative damage scoring
	**Key Metrics**	R^2 = 0.938; MAE = 4.4 axons/sub-image; Bland–Altman LoA = ±14.3 axons	R^2 = 0.944; MAE = 17.7 axons/sub-image; Bland-Altman LoA = [-43.9, 42.8] axons	R = 0.97; Dice = 0.81 ± 0.04; RMSE = 0.044 (normalized); Mean absolute percent difference = 4.4% ± 3.3%	Rat: R^2 = 0.92; Dice = 0.81 ± 0.02; RMSE = 6.18 axons Bland-Altman LoA= [-6.84, 13.57] axons	R^2 = 0.98; RMSE = 63.16; Bland-Altman LoA = [-115.4, 4.83]	R^2 = 0.97; RMSE = 74.71; Bland-Altman LoA = [7.95, 130.87]	N/A
	**Limitations**	Trained on specific staining protocol; performance on other preparations unknown		Validated only on mild-moderate damage; evaluated on subfields only; implementation not publicly available	Cannot reliably segment degenerating axons; brightfield resolution constraints; inter-lab variability in defining normal axons			Cannot segment degenerating/severely damaged axons; image stitching artifacts; border inaccuracies

Goyal et al., 2023a and 2023b describe the same model (AxoNet 2.0) in separate publications focusing on different aspects: 2023a emphasizes cross-species validation and morphometric capabilities, while 2023b focuses on its application to experimental glaucoma models. Goyal et al. 2023b does not report traditional model performance metrics. *Species not explicitly named in Ritch et al. (2020) and in Goyal et al. (2023b); NHP dataset were annotated from Reynaud et al. (2012) from rhesus and cynomolgus macaques from Burgoyne Lab (Devers Eye Institute). **Mouse dataset was annotated from Deng et al. (2021). *Abbreviations*: GAN = generative adversarial network; CNN =convolutional neural network; MAE = mean absolute error; MGS = Morrison grading scale; NHP = non-human primate; ON = optic nerve; RMSE = root mean squared error; TBI =traumatic brain injury; LoA = limits of agreement; PPD = p-paraphenylenediamine.

Supplementary Table S4. Tile-Size Dependency of Performance Metrics

Metric	Tile-Dependent	Rationale
R^2 / Pearson r	Yes	Small tiles restrict count range, inflating correlation
MAE	Yes	Scales with tile axon count magnitude
RMSE	Yes	Scales with tile axon count magnitude
Bias	Yes	Absolute error, not normalized to count
MAPE	No	Percentage error, normalized by count
Relative Bias %	No	Percentage, normalized by count
Dice / IoU / F1	No	Per-pixel overlap ratio
Precision / Recall	No	Per-pixel ratios

Classification of performance metrics by their dependence on evaluation tile dimensions. Tile-dependent metrics vary systematically with the size of image tiles used for evaluation, because smaller tiles restrict the range of axon counts and can inflate correlation values. Tile-independent metrics are normalized to count magnitude or computed as pixel-level ratios, and are therefore comparable across different evaluation scales. No published study of optic nerve axon quantification reported tile-independent counting metrics. *Abbreviations*: MAE = mean absolute error; RMSE = root mean squared error; MAPE = mean absolute percentage error; IoU = intersection over union; F1 = F1 score (harmonic mean of precision and recall).

Supplementary Table S5. Mean of Complete Analysis Metrics for Both Outbred Rat and BXD Mouse datasets

**Dataset**	Outbred Rat (n = 44)			BXD Mouse (n = 74)		
**Model**	AxoNet	AxonDeepSeg	AxoNet 2.0	AxoNet	AxonDeepSeg	AxoNet 2.0
**Pearson r [95% CI]**	0.831 [0.699, 0.903]	0.899 [0.837, 0.939]	0.907 [0.828, 0.949]	0.741 [0.598, 0.861]	0.597 [0.361, 0.774]	0.568 [0.391, 0.692]
**Lin's CCC [95% CI]**	0.169 [0.121, 0.227]	0.478 [0.398, 0.559]	0.649 [0.528, 0.751]	0.724 [0.591, 0.836]	0.478 [0.311, 0.659]	0.496 [0.348, 0.626]
**Spearman rho [95% CI]**	0.854 [0.727, 0.927]	0.922 [0.843, 0.965]	0.909 [0.832, 0.955]	0.66 [0.488, 0.783]	0.506 [0.266, 0.682]	0.525 [0.326, 0.680]
**Spearman p-value**	1.8×10^{-13}	6.0×10^{-19}	1.5×10^{-17}	1.5×10^{-10}	4.3×10^{-6}	1.5×10^{-6}
**MAE [95% CI]**	113.7 [94.2, 135.3]	75.0 [61.0, 90.8]	63.2 [52.5, 76.6]	4,442.5 [3685.5, 5315.4]	7,296.2 [6203.2, 8504.4]	8,038.1 [6903.6, 9236.9]
**RMSE [95% CI]**	133.2 [111.8, 156.5]	90.3 [74.8, 107.0]	74.9 [62.8, 92.9]	5,666.3 [4811.5, 6680.1]	8,902.8 [7763.4, 10249.1]	9,521.3 [8412.2, 10772.2]
**Bias [95% CI]**	-113.7 [-135.3, -94.2]	-75.0 [-90.8, -61.0]	-63.2 [-76.6, -52.5]	+428.6 [-858.2, 1744.0]	-5,081.6 [-6696.1, -3385.0]	+209.9 [-1918.9, 2400.8]
**Reported LoA**	Non-parametric			Parametric		
**Bland-Altman LoA**	[-259.8, -23.8]	[-171.4, -9.2]	[-139.5, -13.5]	[-10,721.1, 11,578.4]	[-19,507.1, 9,343.8]	[-18,574.8, 18,994.5]

**Tile Area (μm^2)**	196	196	196	varies	varies	varies
**px/μm**	18.28	18.28	18.28	2.7	2.7	2.7
**Approx Axons/ Image**	~160	~160	~160	~18,000	~18,000	~18,000
**MAPE (%)**	71.36	49.32	44.29	24.02	39.69	42.62
**Rel. Bias (%)**	-71.36	-49.32	-44.29	+4.07	-23.69	+1.00
**RMSPE (%)**	83.25	56.44	46.81	30.63	48.12	51.47
**Dice**	-	0.29	0.40		N/A	
**IoU**	-	0.18	0.26			
**Precision**	-	0.95	0.94			
**Recall**	-	0.18	0.27			

Abbreviations: Pearson r = correlation between predicted and ground truth counts; CCC = concordance correlation coefficient; CI = confidence interval; LoA = limits of agreement; MAE = mean absolute error; RMSE = root mean squared error; Bias = mean signed difference (positive = overcounting, negative = undercounting); MAPE = mean absolute percentage error (per-image error normalized by ground truth count); Rel. Bias = mean relative bias as percentage of ground truth; IoU = intersection over union. The mouse dataset evaluation tile comprises full optic nerve cross-sections (mean ground truth = 21,443 axons), while the rat dataset comprises 256 x 256 px patches (mean ground truth = 158 axons). GT-normalized metrics (MAPE, Rel. Bias) allow direct comparison across datasets with different count magnitudes. Lin's concordance correlation coefficient (CCC), Spearman's rho, Bland-Altman limits of agreement, and bias-corrected and accelerated (BCa) bootstrap 95% confidence intervals (10,000 resamples) for all metrics in this table are provided. Bland-Altman LoA is shown with its lower and upper limits based on the reported parametric results.

Supplementary Table S6. Shapiro-Wilk normality test results for paired count differences by model and dataset, with the Bland-Altman limits of agreement reporting choice, parametric or nonparametric.

**Dataset**	**Model**	**Shapiro-Wilk W**	**Shapiro-Wilk p**	**Normal at alpha=0.05**	**LoA reporting per Bland-Altman 1999**
Outbred Rats (n = 44)	AxoNet	0.9135	0.00291	No	Non-parametric (2.5/97.5 percentiles)
	AxonDeepSeg	0.8939	0.000708		
	AxoNet 2.0	0.9067	0.00176		
BXD Mice (n = 74)	AxoNet	0.9925	0.945	Yes	Parametric (mean +/- 1.96*SD)
	AxonDeepSeg	0.9674	0.0527		
	AxoNet 2.0	0.9708	0.0842		

Supplementary Table S7. Sensitivity Analysis of Preprocessing Alignment Strategies on Independent Validation Performance on Outbred Rat and BXD Mouse Datasets

Dataset	n	Model	Condition	Pearson r	r 95% CI	Lin's CCC	CCC 95% CI	Spearman rho	rho 95% CI	MAE	MAE 95% CI	RMSE	RMSE 95% CI	Bias	Bias 95% CI	Bias SD	Shapiro-Wilk W	Shapiro-Wilk p	Wilcoxon p (abs vs unmatched)	Wilcoxon median paired delta abs	Wilcoxon p (signed vs un-	TOST equivalence p (5% margin)	Wilcoxon p (abs vs unmatched, Holm-	Wilcoxon p (signed vs un-
Outbred Rat	44	AxoNet	unmatched	0.831	[0.699, 0.903]	0.169	[0.121, 0.227]	0.854	[0.727, 0.927]	113.7	[94.2, 135.3]	133.2	[111.8, 156.5]	-113.7	[-135.3, -94.2]	70.1	0.9135	0.00291						
	44		matched	0.91	[0.826, 0.950]	0.216	[0.166, 0.274]	0.922	[0.856, 0.964]	113.4	[95.5, 133.0]	129.8	[110.8, 151.4]	-113.4	[-133.0, -95.5]	63.9	0.9293	0.00984	0.971	1.95	0.971	7.5×10^{-4}	1.000	1.000
	44		reinhard	0.893	[0.800, 0.942]	0.239	[0.179, 0.308]	0.901	[0.796, 0.955]	107.1	[89.5, 126.6]	124.0	[105.4, 145.2]	-107.1	[-126.6, -89.5]	63.3	0.9225	0.00576	1.0×10^{-8}	-0.72	1.0×10^{-8}	0.733	1.2×10^{-7}	1.0×10^{-7}
	44	ADS	unmatched	0.899	[0.837, 0.939]	0.478	[0.398, 0.559]	0.923	[0.846, 0.965]	74.9	[61.0, 90.8]	90.3	[74.7, 106.9]	-74.9	[-90.8, -61.0]	50.9	0.894	0.000717						
	44		matched	0.874	[0.801, 0.917]	0.375	[0.294, 0.474]	0.912	[0.814, 0.957]	84.5	[68.8, 102.1]	101.5	[84.3, 120.7]	-84.2	[-101.8, -68.2]	57.4	0.9225	0.00579	4.4×10^{-4}	9.5	8.7×10^{-4}	0.988	0.004	0.005
	44		reinhard	0.898	[0.837, 0.937]	0.472	[0.391, 0.554]	0.923	[0.856, 0.963]	74.9	[60.8, 91.1]	90.6	[74.8, 107.5]	-74.9	[-91.1, -60.8]	51.6	0.8914	0.000599	0.512	0.0	0.512	2.2×10^{-7}	1.000	1.000
	44	AxoNet2	unmatched	0.907	[0.828, 0.949]	0.649	[0.528, 0.751]	0.909	[0.832, 0.955]	63.2	[52.5, 76.6]	74.9	[62.8, 92.9]	-63.2	[-76.6, -52.5]	40.6	0.9067	0.00176						
	44		matched	0.904	[0.827, 0.948]	0.505	[0.411, 0.594]	0.912	[0.823, 0.959]	77.4	[64.6, 92.2]	90.4	[76.6, 107.9]	-77.4	[-92.2, -64.6]	47.3	0.904	0.00144	2.0×10^{-8}	9.0	2.0×10^{-8}	1.000	2.2×10^{-7}	1.6×10^{-7}

BXD Mouse									
74	74	74	70	70	70	74	74	74	44
AxoNet2	AxoNet2	AxoNet2	ADS	ADS	ADS	AxoNet			
reinhard	matched	unmatched	reinhard	matched	unmatched	reinhard	matched	unmatched	reinhard
0.564	0.487	0.568	0.629	0.621	0.586	0.687	0.679	0.738	0.897
[0.397, 0.690]	[0.297, 0.629]	[0.403, 0.692]	[0.430, 0.761]	[0.454, 0.732]	[0.377, 0.735]	[0.536, 0.835]	[0.487, 0.836]	[0.597, 0.859]	[0.810, 0.945]
0.492	0.414	0.497	0.57	0.617	0.439	0.655	0.672	0.724	0.618
[0.354, 0.626]	[0.267, 0.541]	[0.359, 0.628]	[0.400, 0.699]	[0.465, 0.734]	[0.289, 0.568]	[0.515, 0.786]	[0.496, 0.816]	[0.592, 0.835]	[0.495, 0.720]
0.522	0.477	0.524	0.559	0.557	0.537	0.586	0.549	0.651	0.903
[0.325, 0.674]	[0.258, 0.642]	[0.326, 0.676]	[0.332, 0.722]	[0.369, 0.698]	[0.313, 0.706]	[0.403, 0.722]	[0.324, 0.714]	[0.480, 0.773]	[0.825, 0.954]
8112.5	8903.0	8034.3	4955.8	4511.8	7264.8	5028.0	4647.0	4388.7	65.4
[6978.4, 9294.3]	[7708.3, 10152.3]	[6885.9, 9215.2]	[4095.9, 5875.7]	[3795.6, 5339.8]	[6162.8, 8498.0]	[4219.9, 5971.5]	[3909.7, 5566.7]	[3662.6, 5235.1]	[53.9, 79.3]
9577.8	10382.0	9518.9	6241.6	5580.5	8790.3	6374.3	5925.0	5632.1	78.0
[8469.5, 10812.2]	[9235.1, 11586.3]	[8399.4, 10762.7]	[5355.2, 7231.0]	[4812.1, 6519.3]	[7657.5, 10121.4]	[5485.1, 7407.1]	[5042.1, 7270.4]	[4814.7, 6618.0]	[65.5, 95.9]
17.3	-2253.2	204.3	-2902.4	705.3	-5505.7	1810.1	-407.4	148.5	-65.4
[-2145.1, 2284.1]	[-4522.1, 151.6]	[-1932.5, 2436.6]	[-4169.4, -1537.8]	[-631.1, 1992.5]	[-7081.1, -3837.2]	[367.7, 3192.8]	[-1651.2, 1060.4]	[-1113.1, 1476.3]	[-79.3, -53.9]
9643.1	10203.7	9581.6	5565.7	5575.7	6901.9	6153.6	5951.3	5668.6	43.0
0.9742	0.9599	0.9708	0.9743	0.9907	0.9819	0.9895	0.9435	0.9893	0.9124
0.134	0.0192	0.0847	0.159	0.887	0.408	0.801	0.00241	0.789	0.00268
0.323	0.057		1.3 × 10^-6	2.3 × 10^-4		0.119	0.540		0.025
2.5	253.0		-553.0	-1596.75		50.17	125.89		1.5
0.015	4.0 × 10^-11		1.2 × 10^-8	8.9 × 10^-11		4.8 × 10^-7	0.035		0.025
1.2 × 10^-6	0.880		1.000	1.000		0.913	0.541		0.173
1.000	0.343		1.3 × 10^-5	0.002		0.594	1.000		0.174
0.073	4.8 × 10^-10		1.1 × 10^-7	9.8 × 10^-10		3.4 × 10^-6	0.106		0.099

Abbreviations: Pearson r = correlation between predicted and ground truth counts; CCC = concordance correlation coefficient; CI = confidence interval; LoA = limits of agreement; MAE = mean absolute error; RMSE = root mean squared error; Bias = mean signed difference (positive = overcounting, negative = undercounting); MAPE = mean absolute percentage error (per-image error normalized by ground truth count); *Wilcoxon.. TOST** . Values are reported for three preprocessing conditions: unmatched (out-of-the-box inference), histogram-matched, and Reinhard-normalized images. Performance metrics include correlation- and agreement-based measures (Pearson r, Lin's CCC, Spearman ϱ), error metrics (MAE, RMSE, Bland–Altman bias), and distributional assessments (Shapiro–Wilk normality testing). Confidence intervals were estimated using 10,000-resample BCa bootstrap methods. Paired Wilcoxon signed-rank tests compare preprocessing conditions against unmatched inference, with Holm–Bonferroni correction applied for multiple comparisons. TOST equivalence testing used a ±5% equivalence margin.
